# Phosphorylation of adducin-1 by cyclin-dependent kinase 5 is important for epidermal growth factor-induced cell migration

**DOI:** 10.1038/s41598-019-50275-0

**Published:** 2019-09-23

**Authors:** Chia-Yi Su, Ruei-Liang Yan, Wen-Hsin Hsu, Ching-Tung Chu, Hsuan-Chia Chang, Chien-Chen Lai, Hui-Ping Hsu, Hong-Chen Chen

**Affiliations:** 1 Department of Life Sciences, National Chung Hsing University, Taichung, Taiwan; 2 Cancer Progression Research Center, National Yang-Ming University, Taipei, Taiwan; 3 Institute of Biochemistry and Molecular Biology, National Yang-Ming University, Taipei, Taiwan; 4 Institute of Molecular Biology, National Chung Hsing University, Taichung, Taiwan; 5 Department of Surgery, National Cheng Kung University Hospital, College of Medicine, National Cheng Kung University, Tainan, Taiwan

**Keywords:** Lamellipodia, Enzyme mechanisms

## Abstract

Cyclin-dependent kinase 5 (Cdk5) is predominantly expressed in neuron and plays an important role in neuronal physiology. Increasing evidence also indicates that Cdk5 may contribute to malignant progression of some types of cancers; however, the underlying mechanism remains elusive. In this study, we found that Cdk5 directly phosphorylated the actin-binding protein adducin-1 (ADD1) at T724 *in vitro* and in intact cells. The capability of the phosphomimetic T724D mutant to bind to actin filaments was lower than that of wild type ADD1 and the T724A mutant. Cdk5 co-localized with ADD1 at the lamellipodia upon epidermal growth factor (EGF) stimulation. The increased lamellipodia formation and cell migration of human breast cancer cells MDA-MB-231 by EGF were accompanied by Cdk5 activation and increased phosphorylation of ADD1 at T724. Depletion of Cdk5 in MDA-MB-231 cells abrogated the effects of EGF on ADD1 T724 phosphorylation, lamellipodia formation, and cell migration. Likewise, depletion of ADD1 suppressed the effects of EGF on lamellipodia formation, cell migration, and invasion, all of which were restored by FLAG-ADD1 WT and the T724D mutant, but not the T724A mutant. Together, our results suggest that phosphorylation of ADD1 at T724 by Cdk5 is important for EGF-induced cell migration and invasion.

## Introduction

Cyclin-dependent kinase 5 (Cdk5), a proline-directed serine/threonine kinase, is an atypical cyclin-dependent kinase that has long been considered as a neuronal kinase^1^. In contrast to other Cdks, Cdk5 is activated by binding to non-cyclin activators p35 and p39, or their respective truncations p25 and p29^2^. Recently, the protein levels of Cdk5 and its activators were found to be aberrantly increased in several types of human tumors^3^. Its kinase activity is important for the migration and metastatic invasion of some forms of cancers, including breast^4^, lung^5^, melanoma^6^, pancreatic^7,8^, and prostate^9^ cancers. Cdk5 modulates neuronal physiology through phosphorylating a variety of microtubule-associated and actin-binding proteins^10,11^. Cdk5 may regulate cancer cell motility and invasion through remodeling of the cytoskeleton^12,13^. Indeed, knockdown of Cdk5 was found to impair actin remodeling in breast cancer cells and melanoma cells^4,6^; however, the molecular mechanisms remain poorly understood.

Adducin is an actin-binding protein that is localized at actin-spectrin junctions^14,15^. The adducin family comprises three isoforms that are encoded by closely related genes including α- (ADD1), β- (ADD2), and γ-adducin (ADD3). ADD1 and ADD3 are expressed in most tissues^15,16^; however, the ADD2 is enriched in the central nervous system and red blood cells^17^. As an actin-binding protein, adducin has three main functions in regulating the actin cytoskeleton: (1) capping the barbed ends of actin filaments (F-actin)^18^, (2) bundling F-actin^19,20^, and (3) recruiting spectrin to F-actin^14,21,22^. All adducin proteins contain similar domain structures consisting of an amino-terminal head domain, a neck region, and a carboxyl-terminal protease-sensitive tail domain^16,23^. The head domain of adducin has been proposed to be important for it to form dimers or oligomers^15,22^. Adducin interacts with spectrin and F-actin through the myristoylated alanine-rich C kinase substrate (MARCKS)-related motif in the extreme C-terminal region of the tail domain^15,16,24^. Phosphorylation of adducin in the MARCKS-related motif by protein kinase C (PKC) decreases its ability to bind spectrin and F-actin^25–27^.

Adducin has multiple cellular functions, which plays important roles in establishing erythrocyte membrane-skeleton^17^, stabilizing cell-cell junctions^28,29^, regulating neuronal synapse plasticity^30,31^, maintaining proper mitotic spindle apparatus during mitosis^32,33^, and facilitating cell migration^34,35^. Adducin has also been implicated in cancer cell migration and metastasis^36–38^. We have previously shown that phosphorylation of ADD1 at S726 by protein kinase Cδ (PKCδ) promotes cell motility^35^. In this study, we demonstrate that Cdk5-mediated phosphorylation of ADD1 at T724 promotes epidermal growth factor (EGF)-induced cell migration and invasion.

## Results

### Cdk5 directly phosphorylates ADD1 at T724

**Figure 1:**
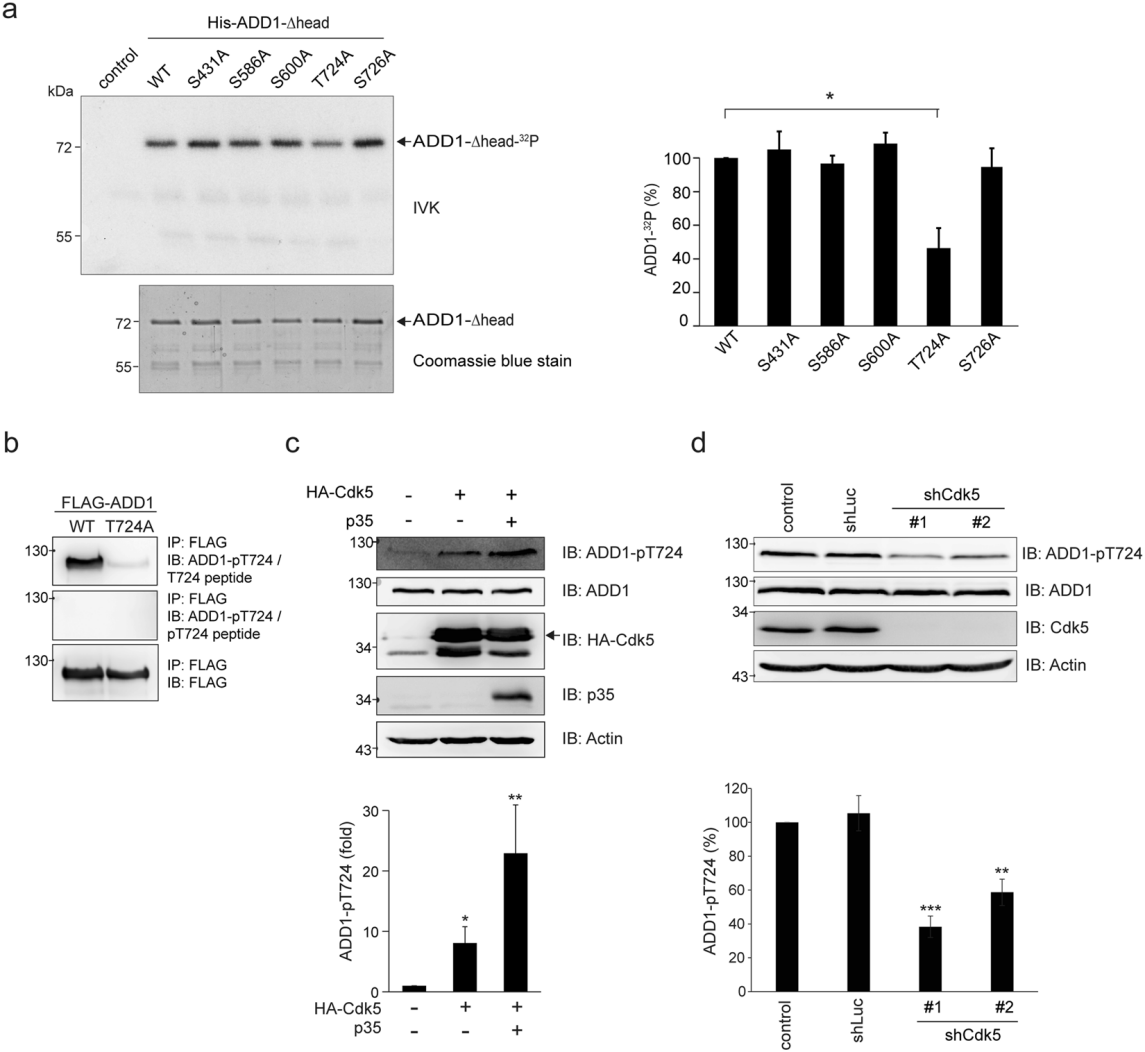
Cdk5 directly phosphorylates ADD1 at T724. (**a**) His-ADD1-Δhead proteins were purified and served as substrates for Cdk5/p35 complex in the *in vitro* kinase assay (IVK). The ^32^P-incorporated proteins were fractionated by SDS-PAGE and visualized by autoradiography. An equal loading of the substrate His-ADD1-Δhead was confirmed by Coomassie blue staining (lower). The phosphorylation of His-ADD1-Δhead was measured and expressed as percentage relative to the WT. Values (means ± s.d.) are from three independent experiments. *P < 0.05. (**b**) FLAG-ADD1 WT or T724A was transiently expressed in HEK293 cells. FLAG-ADD1 was immunoprecipitated with anti-FLAG antibody and the immunocomplexes were analyzed by immunoblotting with anti-FLAG and anti-ADD1 pT724 in the presence of 7 μM T724 phosphopeptide (pT724 peptide) or control T724 peptide. Note that the pT724 peptide successfully blocks the signal using anti-ADD1 pT724. (**c**) HA-Cdk5 was transiently co-expressed with (+) or without (−) its activator p35 in HEK293 cells. An equal amount of the whole cell lysates was analyzed by immunoblotting with antibodies as indicated. The level of ADD1-pT724 was measured and expressed as fold relative to the control in the absence of Cdk5 and p35. Values (means ± s.d.) are from three independent experiments. *P < 0.05; **P < 0.01. (**d**) MDA-MB-231 cells were infected with lentiviruses expressing shRNAs to Cdk5 (shCdk5 #1 and #2) or luciferase (shLuc) as a control. An equal amount of the whole cell lysates was analyzed by immunoblotting with antibodies as indicated. The level of ADD1-pT724 was measured and expressed as percentage relative to the control. Values (means ± s.d.) are from three independent experiments. **P < 0.01; ***P < 0.001.

Our *in silico* analysis suggested that Cdk5 may phosphorylate ADD1 at S431, S600, and T724 (Supplementary Table S1). To validate these putative phosphorylation sites, Cdk5 was overexpressed in HEK293 cells and the phosphorylation of endogenous ADD1 was analyzed by mass spectrometry. Our mass spectrometry analysis revealed that Cdk5 overexpression induced ADD1 phosphorylation at S431, S586, S600 and T724 (Supplementary Table S2). Indeed, Cdk5 directly phosphorylated purified His-tagged ADD1 *in vitro* (Fig. 1a). Mutation of ADD1 at T724 caused ~60% decrease in the phosphorylation by Cdk5 (Fig. 1a), indicating T724 is the major phosphorylation site for Cdk5. To facilitate the detection of T724-phosphorylated ADD1, an antibody (anti-ADD1 pT724) specific to ADD1 pT724 was generated (Fig. 1b). The specificity of this antibody was confirmed by successfully blocking it with a phosphopeptide corresponding to the Thr724 flanking sequences (Fig. 1b). Co-expression of HA-tagged Cdk5 (HA-Cdk5) and its activator p35 apparently increased ADD1 pT724 in HEK293 cells (Fig. 1c). Depletion of Cdk5 by short-hairpin RNA (shRNA) in breast cancer cells MDA-MB-231 significantly diminished ADD1 T724 phosphorylation (Fig. 1d). These results indicate that Cdk5 mainly phosphorylates ADD1 at T724.

### Phosphorylation of ADD1 at T724 may decrease its F-actin binding capability

**Figure 2:**
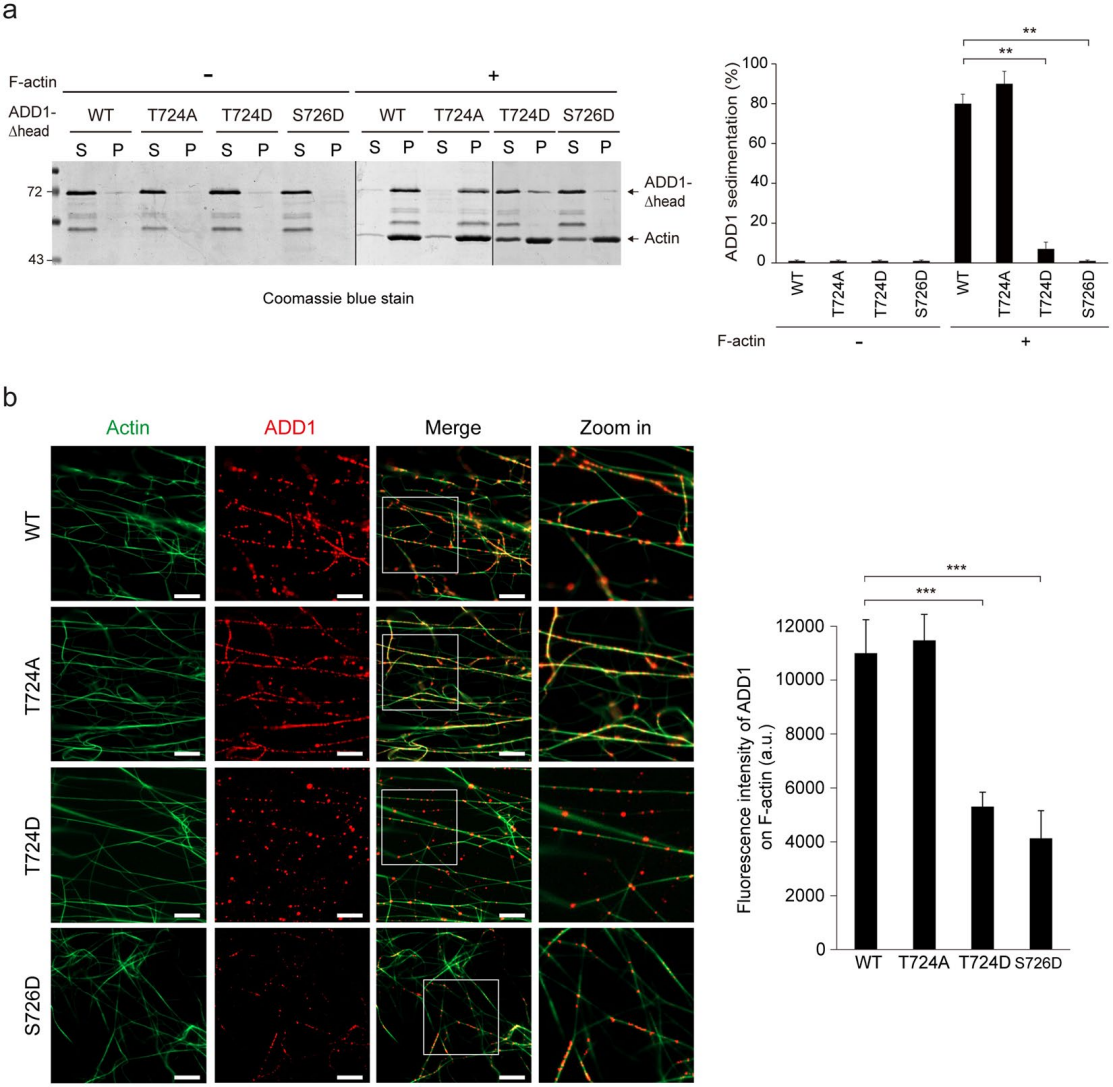
Phosphorylation of ADD1 at T724 may reduce its F-actin binding affinity *in vitro*. (**a**) Co-sedimentation of ADD1 with F-actin *in vitro*. Purified His-ADD1-Δhead proteins were incubated with (+) or without (−) polymerized actins at 25 °C for 30 min and then subjected to centrifugation at 150,000 × g for 20 min. The proteins in the supernatant (S) and pellet (P) fractions were fractionated by SDS-PAGE and stained by Coomassie blue. The percentage of His-ADD1-Δhead ADD1 in the pellet fraction was measured. Values (means ± s.d.) are from three independent experiments. **P < 0.01; ***P < 0.001. (**b**) Purified His-ADD1-Δhead proteins were incubated with F-actin at 25 °C for 30 min and stained for His-ADD1-Δhead and F-actin with anti-ADD1 and Alexa Fluor 488-Phalloidin for 2 h then dropped and fixed on coverslips. An aliquot (10 μl) was dropped onto a coverslip, semidried at 37 °C, and visualized with a Zeiss ApoTome2 system. The Fluorescence intensity of ADD1 per 10-μm F-actin was measured (n ≥ 100). Values (means ± s.d.) are from three independent experiments. ***P < 0.001. a.u., arbitrary unit. Bars, 10 μm.

It is known that phosphorylation of ADD1 in the MARCKS-related domain inhibits its F-actin binding ability^25–27^; therefore, we examine whether ADD1 T724 phosphorylation affects its F-actin binding capability. His-tagged ADD1 with a deletion of the head domain (His-ADD1-Δhead) was purified and subjected to F-actin co-sedimentation experiment. In the absence of F-actin, purified His-ADD1 and the mutants were soluble and retained in the supernatant (Fig. 2a). In the presence of F-actin, ADD1 WT and the T724A mutant were co-sediment with F-actin and detected in the pellet, whereas the phosphomimetic mutant (T724D) was retained in the supernatant (Fig. 2a). It has been reported that phosphorylation of ADD1 at S726 diminishes its F-actin binding capability^26,27^. Accordingly, the S726D mutant was not co-sediment with F-actin in our experiments (Fig. 2a). To visualize the interaction between ADD1 and F-actin, purified ADD1 was incubated with F-actin and stained for ADD1 and F-actin with anti-ADD1 antibody and phalloidin, respectively. Our confocal microscopy analysis showed that both T724D and S726D mutants bound to F-actin ~55% less than ADD1 WT and the T724A mutant did (Fig. 2b). These results suggest that the phosphorylation of ADD1 at T724 may diminish its F-actin binding affinity.

### Cdk5-mediated phosphorylation of ADD1 at T724 promotes cell migration and invasion

**Figure 3:**
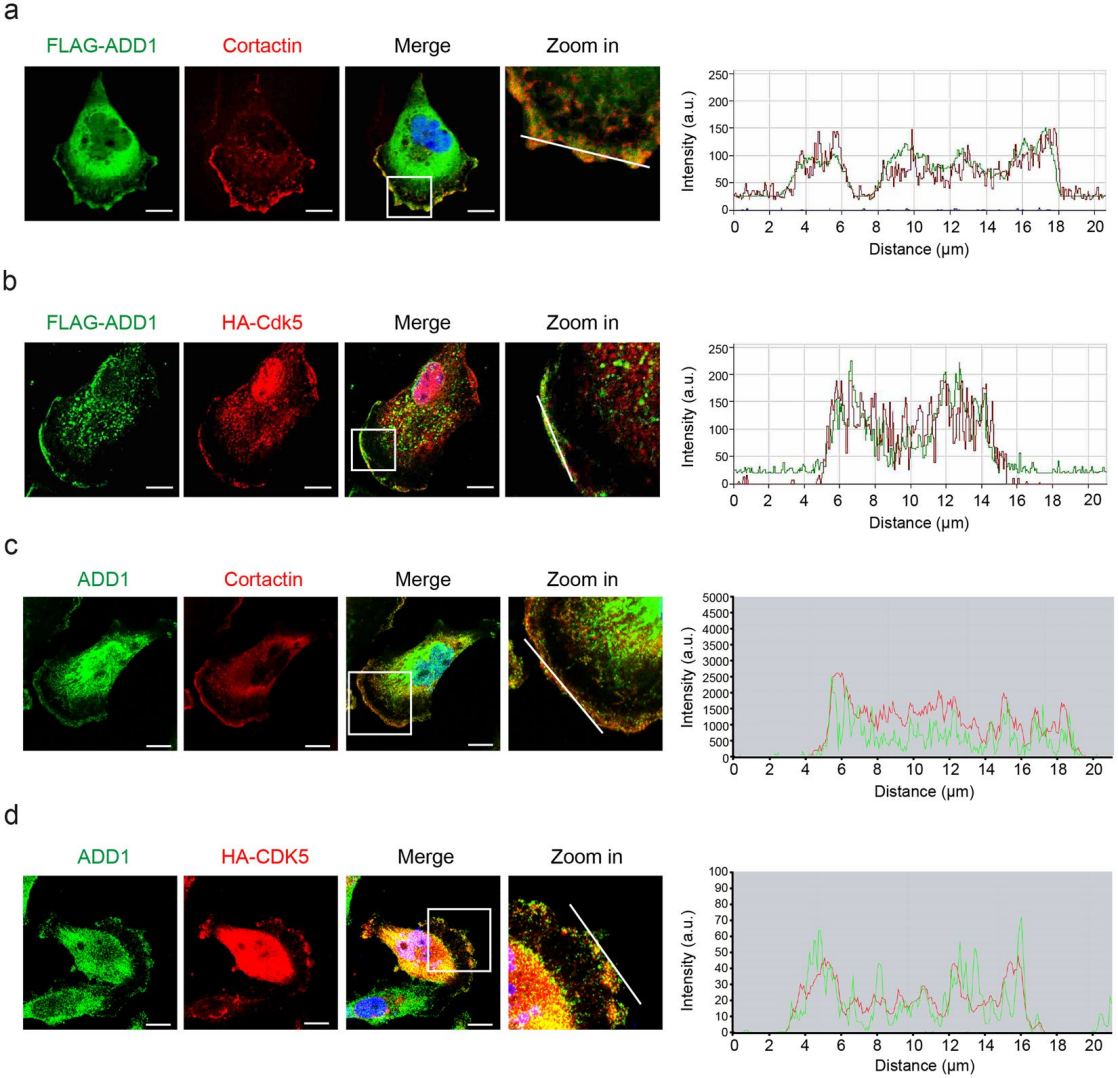
ADD1 colocalizes with Cdk5 at lamellipodia. (**a**) MDA-MB-231 cells transiently expressing FLAG-ADD1 were fixed, stained with anti-FLAG and anti-cortactin (as a marker for lamellipodia), and visualized with a Zeiss LSM510 confocal microscope. Graph shown at right represents the relative fluorescence intensity of the line that was scanned by confocal microscopy. a.u., arbitrary unit. Bars, 10 μm. (**b**) MDA-MB-231 cells transiently co-expressing FLAG-ADD1 and HA-Cdk5 were fixed, stained with anti-FLAG and anti-HA, and visualized with a Zeiss LSM510 confocal microscope. Graph shown at right represents the relative fluorescence intensity of the line that was scanned by confocal microscopy. a.u., arbitrary unit. Bars, 10 μm. (**c**) MDA-MB-231 cells were fixed, stained with anti-ADD1 and anti-cortactin, and visualized with a Zeiss LSM880 confocal microscope. Graph shown at right represents the relative fluorescence intensity of the line that was scanned by confocal microscopy. a.u., arbitrary unit. Bars, 10 μm. (**d**) MDA-MB-231 cells transiently expressing HA-Cdk5 were fixed, stained with anti-ADD1 and anti-HA, and visualized with a Zeiss LSM880 confocal microscope. Graph shown at right represents the relative fluorescence intensity of the line that was scanned by confocal microscopy. a.u., arbitrary unit. Bars, 10 μm.

**Figure 4:**
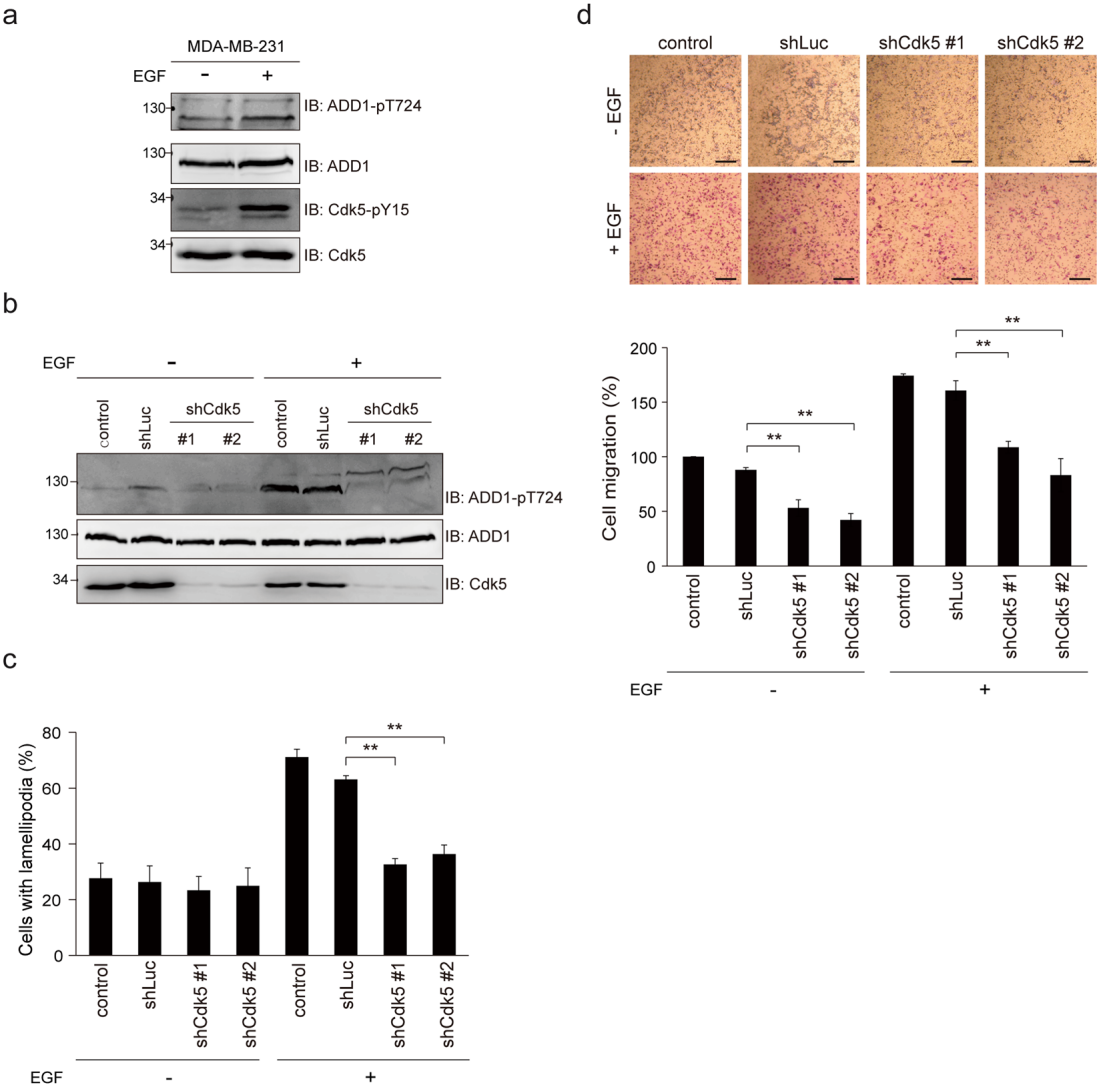
Depletion of CdK5 abrogates the effects of EGF on ADD1 T724 phosphorylation, lamellipodia formation, and cell migration. (**a**) MDA-MB-231 cells were serum-starved for 16 h and treated with (+) or without (−) 40 ng/ml EGF for 15 min. An equal amount of the whole cell lysates was analyzed by immunoblotting with antibodies as indicated. (**b**) MDA-MB-231 cells were infected with lentiviruses expressing shRNAs to Cdk5 (shCdk5 clone#1 and #2) or luciferase (shLuc) as a control. The cells were serum-starved for 16 h and treated with (+) or without (−) 40 ng/ml EGF for 15 min. (**c**) MDA-MB-231 cells described in (**b**) were plated on collagen-coated coverslips, and treated with (+) or without (−) 200 ng/ml EGF for 6 h. The percentage of cells with lamellipodia in the total number of counted cells was measured (n ≥ 200). Values (means ± s.d.) are from three independent experiments. **P < 0.01. (**d**) MDA-MB-231 cells described in (**b**) were suspended in serum-free medium and subjected to the cell migration assay with (+) or without (−) 40 ng/ml EGF in the lower chambers. After 6 h, the migrated cells were fixed, stained, and counted using a light microscope. Representative micrographs are shown. Bars, 100 μm. The number of migrated cells were measured and expressed as percentage relative to the control in the absence of EGF. Values (means ± s.d.) are from three independent experiments. **P < 0.01.

**Figure 5:**
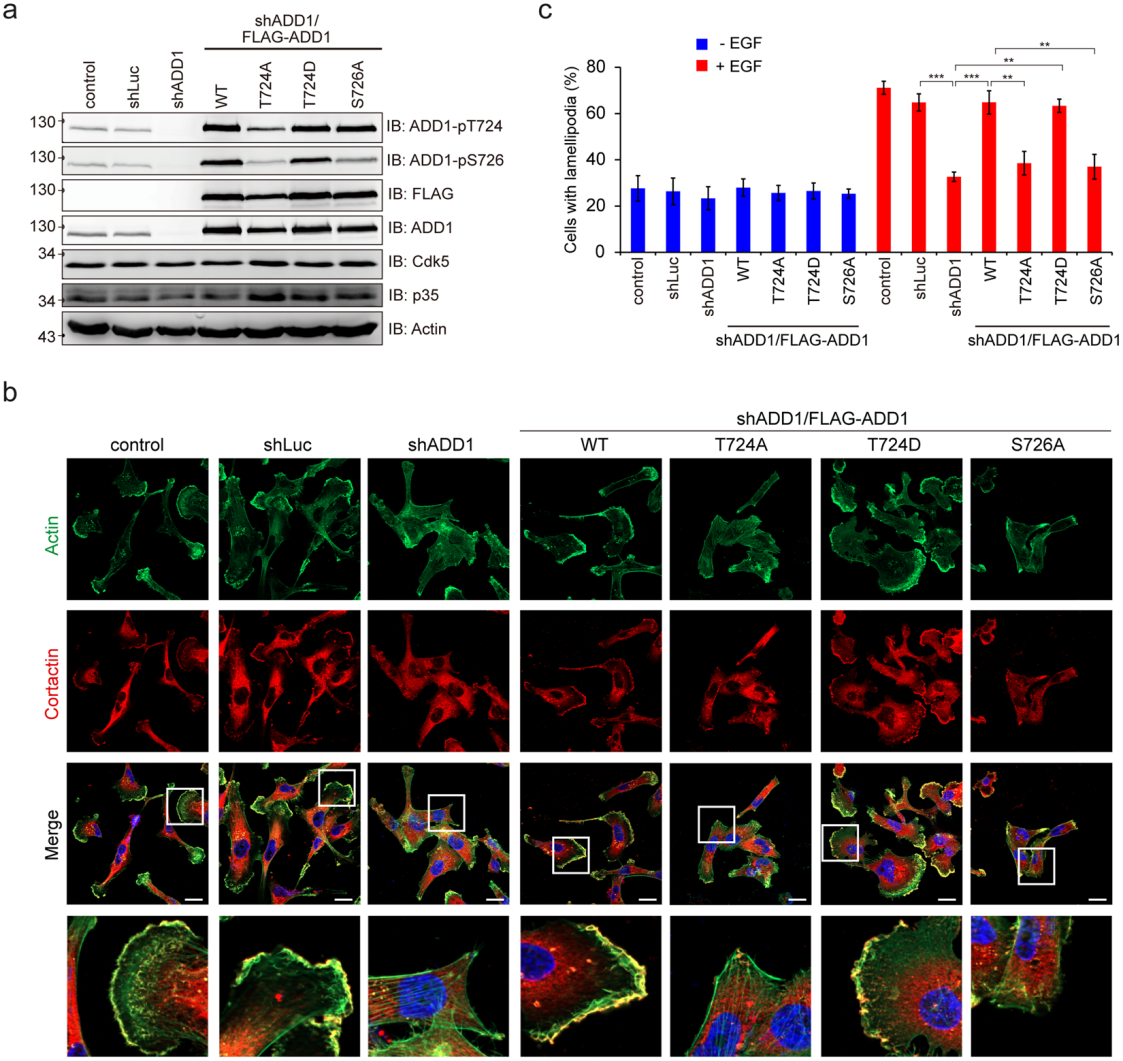
Phosphorylation of ADD1 at T724 is important for lamellipodia formation. (**a**) MDA-MB-231 cells were infected with lentiviruses expressing shRNAs specific to ADD1 (sh-ADD1) or luciferase (shLuc). FLAG-ADD1 or its mutants were re-expressed in the ADD1-depleted cells (sh-ADD1/FLAG-ADD1). The cells were serum-starved for 18 h and treated with 40 ng/ml EGF for 15 min. An equal amount of whole cell lysates was analyzed by immunoblotting with the indicated antibodies. (**b**) The cells as described in (**a**) were plated on collagen-coated coverslips, serum-starved, and treated with (+) or without (−) 200 ng/ml EGF for 6 h. The cells were fixed, stained for cortactin and F-actin, and visualized with a Zeiss ApoTome2 system. Representative micrographs are shown. Bars, 20 μm. (**c**) The percentage of cells with lamellipodia in the total number of counted cells was measured (n ≥ 200). Values (means ± s.d.) are from three independent experiments. **P < 0.01; ***P < 0.001.

**Figure 6:**
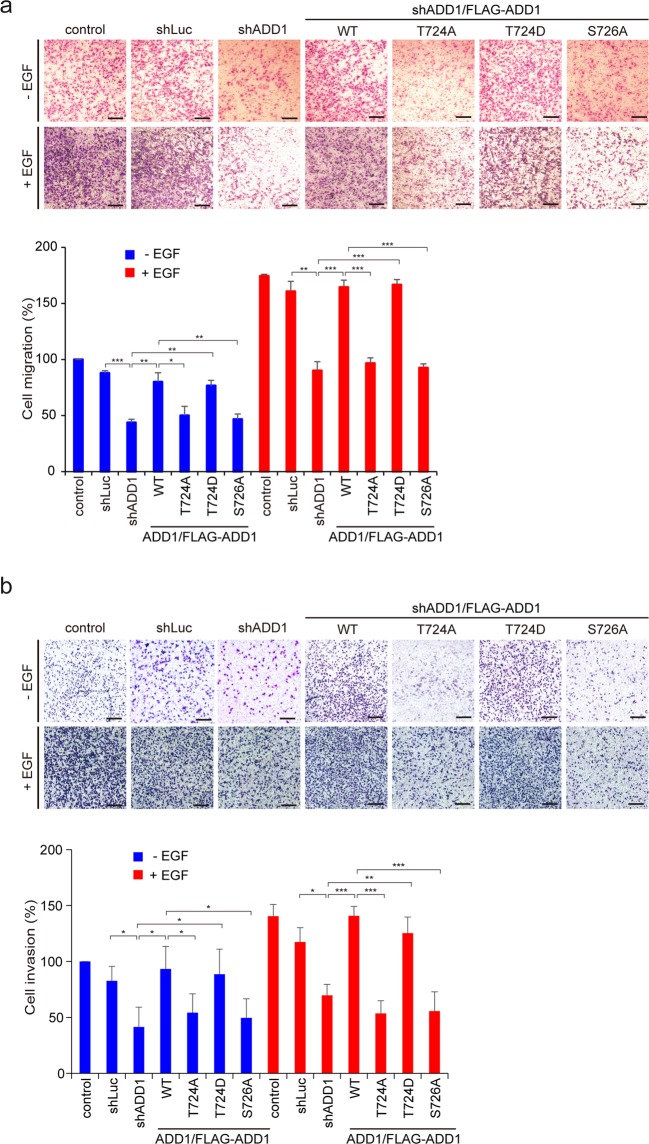
Phosphorylation of ADD1 at T724 is important for cell migration and cell invasion. (**a**) MDA-MB-231 cells were infected with lentiviruses expressing shRNAs specific to ADD1 (sh-ADD1) or luciferase (shLuc). FLAG-ADD1 or its mutants were re-expressed in the ADD1-depleted cells (sh-ADD1/FLAG-ADD1). The cells were suspended in serum-free medium and subjected to the cell migration assay with (+) or without (−) 40 ng/ml EGF in the lower chambers. After 6 h, the migrated cells were fixed, stained, and counted using a light microscope. Representative micrographs are shown. Bars, 100 μm. The number of migrated cells were measured and expressed as percentage relative to the control in the absence of EGF. Values (means ± s.d.) are from three independent experiments. *P < 0.05; **P < 0.01; ***P < 0.001. (**b**) MDA-MB-231 cells described in (**a**) were suspended in serum-free medium and subjected to the invasion assay with (+) or without (−) 40 ng/ml EGF in the lower chambers. After 24 h, the cells that migrated through Matrigel were fixed, stained and counted using a light microscope. Representative micrographs are shown. Bars, 100 μm. The number of cells were measured and expressed as percentage relative to the control in the absence of EGF. Values (means ± s.d.) are from three independent experiments. *P < 0.05; **P < 0.01; ***P < 0.001.

ADD1 has been shown to localize at the leading edge of lamellipodia of migratory cells^35^. In this study, we showed that upon EGF stimulation, ADD1 co-localized with cortactin (as a marker for lamellipodia) and Cdk5 at the lamellipodia of MDA-MB-231 cells (Fig. 3). EGF induced activation of Cdk5, as manifested by increased Tyr15 phosphorylation, which was accompanied by increased ADD1 T724 phosphorylation in MDA-MB-231 cells (Fig. 4a). Depletion of Cdk5 in MDA-MB-231 cells abrogated the effects of EGF on ADD1 T724 phosphorylation (Fig. 4b), lamellipodia formation (Fig. 4c), and cell migration (Fig. 4d). Likewise, depletion of ADD1 apparently inhibited the effects of EGF on lamellipodia formation (Fig. 5), cell migration (Fig. 6a), and invasion (Fig. 6b). These defects were restored by re-expression FLAG-ADD1 and T724D mutant, but not T724A or S726A mutant (Figs 5 and 6). These results suggest that phosphorylation of ADD1 at both T724 and S726 may be important for EGF-induced cell migration and invasion.

### Increased expression of ADD1 and its phosphorylation at T724 and S726 may be correlated with tumor malignancy

**Figure 7:**
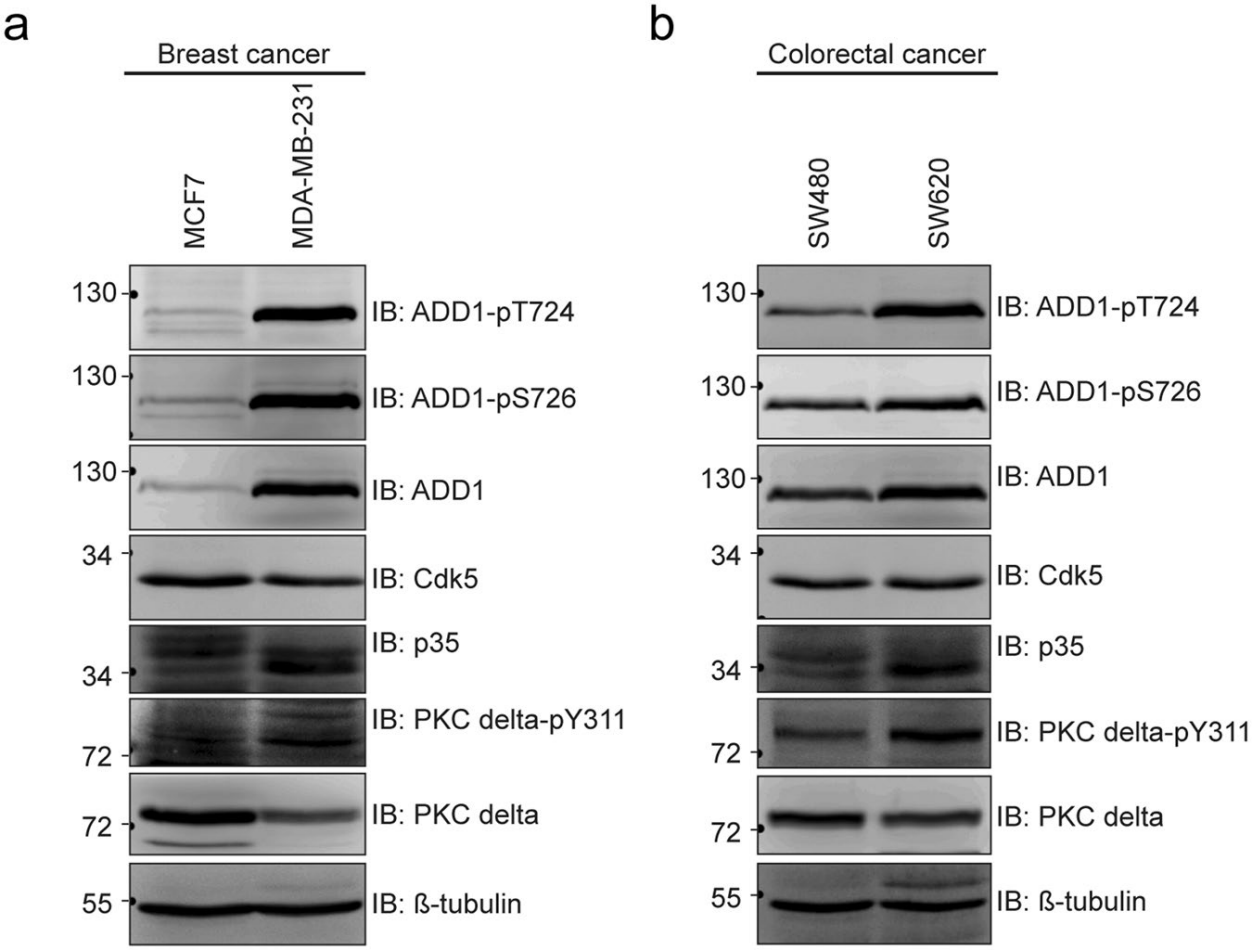
Increased expression of ADD1 and its phosphorylation at T724 and S726 may be correlated with tumor malignancy. (**a**) An equal amount of whole cell lysates from high metastatic MDA-MB-231 and low metastatic MCF7 breast cancer cells was analyzed with immunoblotting with antibodies as indicated. (**b**) An equal amount of whole cell lysates from high metastatic SW620 and low metastatic SW480 colorectal cancer cells was analyzed with immunoblotting blotting with antibodies as indicated.

Increasing evidence indicates that the expression levels of Cdk5 and its activators are increased in several types of cancers^3^. We found that the expression levels of p35 and ADD1 were higher in metastatic breast cancer cells MDA-MB-231 than in non-metastatic breast cancer cells MCF7 (Fig. 7a). Likewise, metastatic colorectal cancer cells SW620 showed higher expression of p35 and ADD1 than non-metastatic colorectal cancer cells SW480 did (Fig. 7b). We have previously shown that phosphorylation of ADD1 at S726 by PKCδ promotes cell motility^35^. We found in this study that the activation of PKCδ was also elevated in highly metastatic cancer cell lines (Fig. 7a,b). The increased expression of ADD1 was correlated with increased phosphorylation at T724 and S726. These results suggest that activation of Cdk5 and PKCδ and phosphorylation of ADD1 at T724 and S726 may be correlated with the metastatic potential of cancer cells.

## Discussion

Cdk5 has previously been shown to promote cell migration upon EGF stimulation^39^; however, the underlying mechanism is not clear. In this study, we identified ADD1 as a novel substrate of Cdk5 (Fig. 1) and demonstrated that ADD1 phosphorylation at T724 by Cdk5 is important for EGF-induced cell migration and invasion in MDA-MB-231 breast cancer cells (Fig. 6). ADD1 is known to bundle and cap F-actin at the barbed ends^18,20^. Our results suggest that phosphorylation of ADD1 at T724 may diminish its F-actin binding affinity (Fig. 2), which may thereby allow F-actin to expose their barbed ends, leading to elongation and remodeling of F-actin. We found that phosphorylation of ADD1 at T724 and S726, both of which are located in the MARCKS-related domain, were increased upon EGF stimulation (Fig. 5a). Therefore, it is possible that the phosphorylation of ADD1 at both residues may facilitate dynamic remodeling of the actin cytoskeleton during cell migration upon EGF stimulation. This notion is supported partially by ADD1 co-localization with F-actin at the leading edge of the lamellipodia of migratory cells (Fig. 3).

Cdk5 and its activator p35 expression have been shown to be upregulated in breast cancer cells^4^ and correlated with tumor progression and poor prognosis^40,41^. In addition, depletion of Cdk5 was found to impair actin remodeling in breast cancer cells^4^. In this study, we found that in MDA-MB-231 cells, Cdk5 was activated upon EGF stimulation (Fig. 4a) and the depletion of Cdk5 in those cells efficiently abrogated the effects of EGF on lamellipodia formation and cell migration (Fig. 4b–d). Our and others results together suggest that Cdk5 may be a critical effector to transmit the EGF signaling, leading to reorganization of the actin cytoskeleton and cell migration in cancer cells. Moreover, we found that the expression of Cdk5 activator p35 and ADD1 were much higher in metastatic breast cancer cells MDA-MB-231 than in non-metastatic breast cancer cells MCF7 (Fig. 7a), suggesting that aberrant expression of Cdk5 and/or its activator p35 as well as elevated ADD1 phosphorylation at T724 may serve as biomarkers for prognosis of breast cancers. However, this possibility remains to be examined in samples from breast cancer patients.

In this study, we show that Cdk5 phosphorylates ADD1 at T724. The T724 and flanking amino acids are well conserved in ADD2 and ADD3. Because both Cdk5 and ADD2 are enriched in the nervous system, it will be of interest to examine whether Cdk5 phosphorylates ADD2 under physiological or pathological conditions. In fact, it was reported that prior priming phosphorylation of ADD2 by Cdk5 enhances its phosphorylation by glycogen synthase kinase 3, which is important for neurite outgrowth in neurons^42^. Moreover, Cdk5 was shown to phosphorylate the Wiskott-Aldrich syndrome protein-family verprolin homologous protein 1 (WAVE1) and regulate actin polymerization in neurons^43,44^. The possibility of whether Cdk5 targets WAVE proteins to promote lamellipodia in cancer cells or other non-neuronal cells remains to be tested.

We have previously shown that phosphorylation of ADD1 at S726 by PKCδ promotes cell migration^35^. It is worth noting that the T724A mutant was less phosphorylated at S726 upon EGF stimulation (Fig. 5a), suggesting that prior priming phosphorylation of ADD1 at T724 by Cdk5 may be important for its phosphorylation at S726 by PKCδ. These results together suggest that Cdk5 and PKCδ may coordinately regulate F-actin organization and promote cancer cell migration through ADD1. Indeed, elevated activation of Cdk5 and PKCδ and increased phosphorylation of ADD1 at T724 and S726 were detected in the highly metastatic cancer cells (Fig. 7). In summary, we demonstrate that Cdk5 directly phosphorylates ADD1 at T724, which is important for cell migration and invasion. This work not only unveils the mechanism of Cdk5 in regulating cell migration and invasion through ADD1, but also highlights the role of ADD1 in cancer progression.

## Materials and Methods

### Materials

The rabbit polyclonal antibody specific to ADD1 pT724 was generated using synthesized phospho-peptides KLH-CKKKKFRTpPSFLKKS (pT724 peptide) as the antigen (AllBio, Inc., Taichung, Taiwan). The rabbit polyclonal anti-ADD1 pS726 (sc-16736), anti-ADD1 (sc-25731), anti-PKCδ (sc-937), and anti-cortactin (sc-11408) antibodies and mouse monoclonal anti-Cdk5 (sc-249) and anti-β-tubulin (sc-5274) antibodies were purchased from Santa Cruz Biotechnology. The rabbit polyclonal anti-FLAG (F7425) and anti-Cdk5 pY15 (SAB4504276) antibodies and mouse anti-β-actin (A5441) antibody were purchased from Sigma-Aldrich. The rabbit monoclonal anti-p35 (C64B10) antibody and rabbit polyclonal anti-PKCδ pY311 were purchased from Cell Signaling Technology. The mouse monoclonal anti-HA (MMS-101P) antibody was purchased from Covance. The HRP-conjugated goat anti-rabbit and goat anti-mouse antibodies were purchased from Jackson ImmunoResearch Laboratories, Inc. Dulbecco’s modified Eagle’s medium (DMEM), Alexa Fluor 488- and Alexa Fluor 546-conjugated secondary antibodies, and Lipofectamine were purchased from Invitrogen Life Technologies. Purified Cdk5/p35 was purchased from EMD Millipore. EGF was purchased from R&D Systems. G418 and puromycin were purchased from Merck. Matrigel was purchased from BD Transduction Laboratories. The muscle actin was purchased from Cytoskeleton, Inc.

### Plasmids

The plasmid pCMV-3Tag-3A-ADD1 WT for FLAG-ADD1 WT was constructed in our laboratory and described previously^32^. Adducin mutants including S431A, S586A, S600A, T724A, T724D, S726A, and S726D were generated by using a site-directed mutagenesis kit (QuikChange, Agilent Technologies) and was confirmed by dideoxy DNA sequencing. To express the FLAG-ADD1 WT, T724A, T724D, or S726D mutant by lentiviral infection, the corresponding cDNAs were PCR amplified using pCMV-3Tag-3A-ADD1 WT, T724A, T724D, or S726D as the template and then subcloned into the lentiviral vector pLAS3w.Pneo or pLAS3w.Phyg (National RNAi Core Facility, Academia Sinica, Taipei, Taiwan). To construct the plasmid encoding the His-tagged ADD1 ΔHead domain (aa 350–737), cDNAs from pCMV-3Tag-3A-ADD1 ΔHead was subcloned into pET-21d vector (EMD Millipore). The pEGFP-C3-Cdk5 and pcDNA3-p35 were gifts from Dr. Ho Lin (National Chung Hsing University, Taichung, Taiwan). The full-length Cdk5 cDNA was cloned into the BamHI and XbaI sites of the pcDNA3.1(+)-HA(3) vector.

### Cell culture and transient transfection

MDA-MB-231, MCF7, SW480, SW620, and HEK293 cells were obtained from American Type Culture Collection and maintained in DMEM supplemented with 10% fetal bovine serum (Invitrogen). Cell culture and transient transfection were performed as described previously^32^.

### Lentiviral production and infection

The lentiviral expression system, consisting of the pLKO-AS1-puromycin (puro) plasmid encoding shRNAs, the pLAS3w.Phyg plasmid, and the pLAS3w.Pneo plasmid, were obtained from the National RNAi Core Facility (Academia Sinica, Taiwan). The target sequence for ADD1 was 5′-GCAGAATTTACAGGACATTAA-3′. The target sequences for Cdk5 were 5′-CCTGAGATTGTAAAGTCATTC-3′ (#1) and 5′-CAGAACCTTCTGAAGTGTAAC-3′ (#2). For FLAG-ADD1 WT, T724A, T724D, and S726D expression, FLAG-ADD1 cDNAs were amplified by PCR and then subcloned into the pLAS3w.Pneo vector. For FLAG-ADD1 S726D expression, FLAG-ADD1 S726D cDNA was amplified by PCR and then subcloned into the pLAS3w.Phyg vector. Lentiviral production and infection were performed as described previously^32^.

### Immunoblotting and immunoprecipitation

To prepare whole-cell lysates, cells were lysed with 1% NP-40 lysis buffer (1% NP-40, 20 mM Tris-HCl pH 8.0, 137 mM NaCl, 10% glycerol, and 1 mM Na_3_VO_4_) containing protease inhibitor cocktail (Roche). Immunoblotting and immunoprecipitation were performed as described previously^33^.

### *In vitro* kinase assay

His-tagged ADD1-Δhead and mutants were expressed in E. coli and purified by chelating Sepharose (Amersham Biosciences, NJ) according to the manufacturer’s instructions. Kinase reactions were carried out in 40 μl of kinase buffer (25 mM Tris-HCl, pH7.4, 10 mM MgCl_2_) containing 10 μCi of γ-^32^P-ATP (3000 Ci mmol^−1^; PerkinElmer Life Sciences), 10 ng Cdk5/p35, and purified 0.5 μg His-ADD1 proteins at 25 °C for 20 min. Reactions were terminated by addition of SDS sample buffer, and the ^32^P-incorporated proteins were fractionated by SDS-PAGE and visualized by autoradiography. The radioisotope activity was quantified using a phosphoimager system (Pharmacia).

### F-actin co-sedimentation assay

Purified muscle actin (Cytoskeleton, Inc.) at 0.16 μg/μl in the general actin buffer (5 mM Tris-HCl, pH8.0; 0.2 mM CaCl_2_) was allowed to polymerize by addition of 1/10 volume (2.5 μl) of the polymerization buffer (500 mM KCl; 20 mM MgCl_2_; 10 mM ATP) at 25 °C for 60 min. For F-actin co-sedimentation assay, purified His-ADD1-Δhead proteins (final concentration: 0.08 μg/μl) were incubated with F-actin at 25 °C for 30 min and then subjected to centrifugation at 150,000 × g for 20 min at 4 °C. The supernatants were collected and the pellets were dissolved in distilled water. His-ADD1-Δhead proteins in an equal volume of the supernatant and pellet fractions were fractionated by SDS-PAGE, visualized with Coomassie blue stain, and measured using ImageJ software.

### Immunofluorescence staining and image analysis

To visualize the binding of ADD1 to F-actin, purified His-ADD1-Δhead proteins (final concentration: 0.04 μg/μl) were incubated with F-actin at 25 °C for 30 min. The mixtures were stained with anti-ADD1 (1:100) and Alexa Fluor 488-Phalloidin (1:100) for 2 h, and followed by Alexa Fluor 546-conjugated secondary antibody for another 2 h. An aliquot (10 μl) was dropped onto a coverslip, semidried at 37 °C, mounted in Anti-Fade Dapi-Fluoromount-G (SouthernBiotech), and visualized with a Zeiss ApoTome2 system. The Fluorescence intensity of ADD1 per 10-μm F-actin was measured using ZEN software.

Cells were plated on 10 μg/ml collagen-coated glass coverslips for 24 h, fixed with phosphate-buffered saline containing 4% paraformaldehyde for 30 min and then permeabilized with 0.1% Triton X-100 for 15 min at room temperature. The fixed cells were stained with primary antibodies at room temperature for 2 h and then incubated with Alexa Fluor 488-, or 546-conjugated secondary antibodies (Invitrogen) for 2 h. Coverslips were mounted on the slides with mounting medium (Anti-Fade Dapi-Fluoromount-G, Southern Biotech). The images in Fig. 3 were acquired using a laser-scanning confocal microscope imaging system (Carl Zeiss) with a Plan Apochromat 100x/NA 1.4 oil immersion objective (Carl Zeiss). The images in Figs 2b and 5c were acquired using a Zeiss ApoTome2 system equipped with Plan Apochromat 40x/NA 1.3 and 100x/NA 1.4 oil immersion objectives and a camera (ORCA-Flash4.0 V2; Hamamatsu).

### Cell migration assay

Cell migration assay was performed as described previously^45^. In brief, the medium containing 10 μg/ml type I collagen was added to the lower chamber. The lower and upper chambers were separated by a polycarbonate membrane with 8-μm pores (Poretics, Livermore, CA). 5 × 10^3^ cells in 50 μl serum-free medium were loaded into upper chamber. Cells were allowed to migrate for 6 h at 37 °C in a humidified atmosphere containing 5% CO_2_. The membrane was fixed in methanol for 10 min and stained with modified Giemsa stain (Sigma-Aldrich) for two h. Cells on the upper side of the membrane were removed by cotton swabs. Cells on the lower side of the membrane were counted under a light microscope.

### Cell invasion assay

Cell invasion assay was performed as described previously^45^. The lower chamber was loaded with 0.75 ml DMEM with 10% serum. 5 × 10^4^ cells in 0.25 ml of serum-free medium were added to the upper chamber. After 24 h, the cells that had migrated through the Matrigel were fixed by methanol, stained by Giemsa stain, and counted.

### Mass spectrometry

Cdk5 was transiently overexpressed in HEK293 cells, and endogenous ADD1 was immunoprecipitated by anti-ADD1. The immunocomplexes were fractionated by SDS-PAGE and stained with Coomassie blue. Mass spectrometry to identify the phosphorylation sites was performed as described previously^32^.

### Statistics

Significance was determined by unpaired Student’s t-test for two samples. Error bars represent standard deviation (s.d.). The significance levels are indicated by asterisks: *P < 0.05, **P < 0.01, and ***P < 0.001.

## Supplementary information

Supplementary Information
