# Supplementary material for: Phosphorylation of adducin-1 by cyclin-dependent kinase 5 is important for epidermal growth factor-induced cell migration
Source: Sci Rep. 2019 Sep 23;9:13703. doi: 10.1038/s41598-019-50275-0 (PMC6757057; doi:10.1038/s41598-019-50275-0)

Supplementary Information

# Phosphorylation of adducin-1 by cyclin-dependent kinase 5 is important for epidermal growth factor-induced cell migration

Chia-Yi Su, Ruei-Liang Yan, Wen-Hsin Hsu, Ching-Tung Chu, Hsuan-Chia Chang, Chien-Chen Lai, Hui-Ping Hsu, and Hong-Chen Chen

*Corresponding author. Email: hcchen1029@ym.edu.tw

**Supplementary Table S1**. Predicted ADD1 phosphorylation sites for Cdk5.

| Software | ADD1 |
|---|---|
| Scansite[1] | S431 |
| | S600 |
| | T724 |
| GPS[2] | S431 |
| | S600 |
| | T724 |

**Supplementary Table S2**. Identification of ADD1 phosphorylation sites by mass spectrometry analysis.

| ADD1 | Cdk5[a] | |
|---|---|---|
| domain | - | + |
| head | S12 | S12 |
| neck | S355 | S355 |
| | S358 | S358 |
| | | S431 |
| | S465 | S465 |
| tail | | S586 |
| | | S600 |
| | | T724 |
| | S726 | S726 |

a. HEK293 cells were transiently transfected with (+) or without (-) Cdk5. Endogenous ADD1 was immunoprecipitated with anti-ADD1 antibody and the phosphorylation sites of ADD1 were identified by mass spectrometry.

## References


1. Obenaur, J.C., Cantley, L.C. & Yaffe, M.B. Scansite 2.0: proteome-wide prediction of cell signaling interactions using short sequence motifs. *Nucleic Acids Res.* **31,** 3635-3641; http://scansite.mit.edu (2003).
2. Xue, Y. *et al.* GPS 2.0, a tool to predict kinase-specific phosphorylation sites in hierarchy. *Mol. Cell Proteomics* **7,** 1598-1608; https://omictools.com/gps-2-tool (2008).

# Raw data

## Supple. Fig. 1(a)

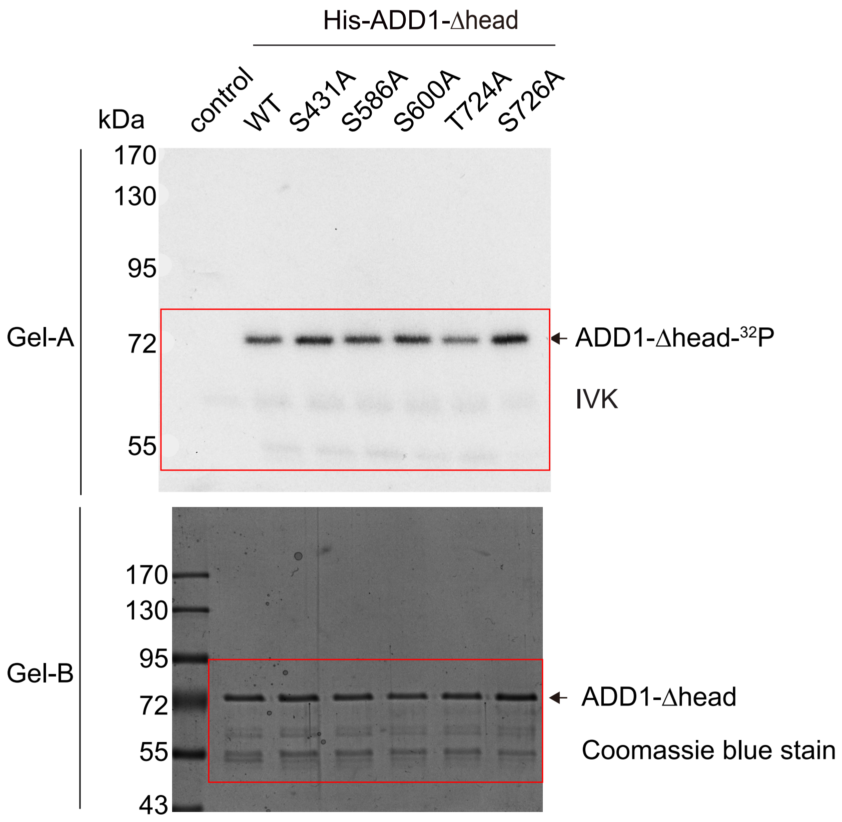


## Supple. Fig. 1(b)

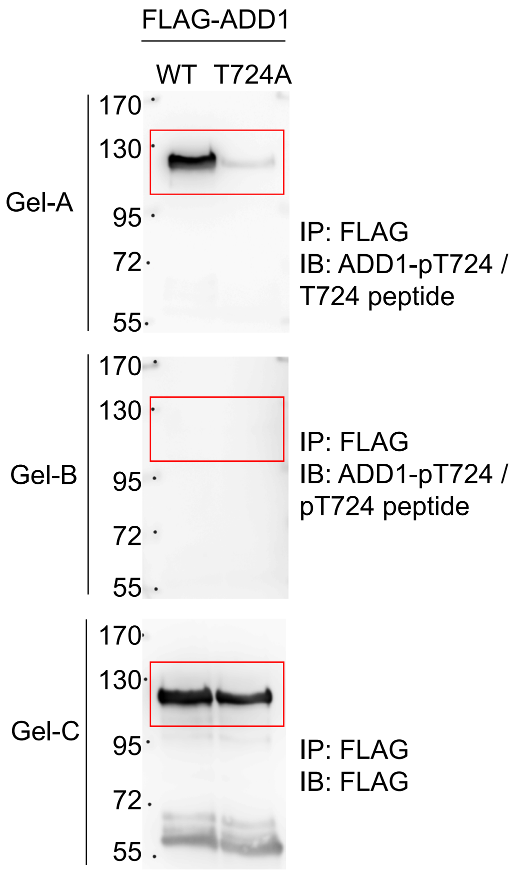

## Supple. Fig. 1(c)

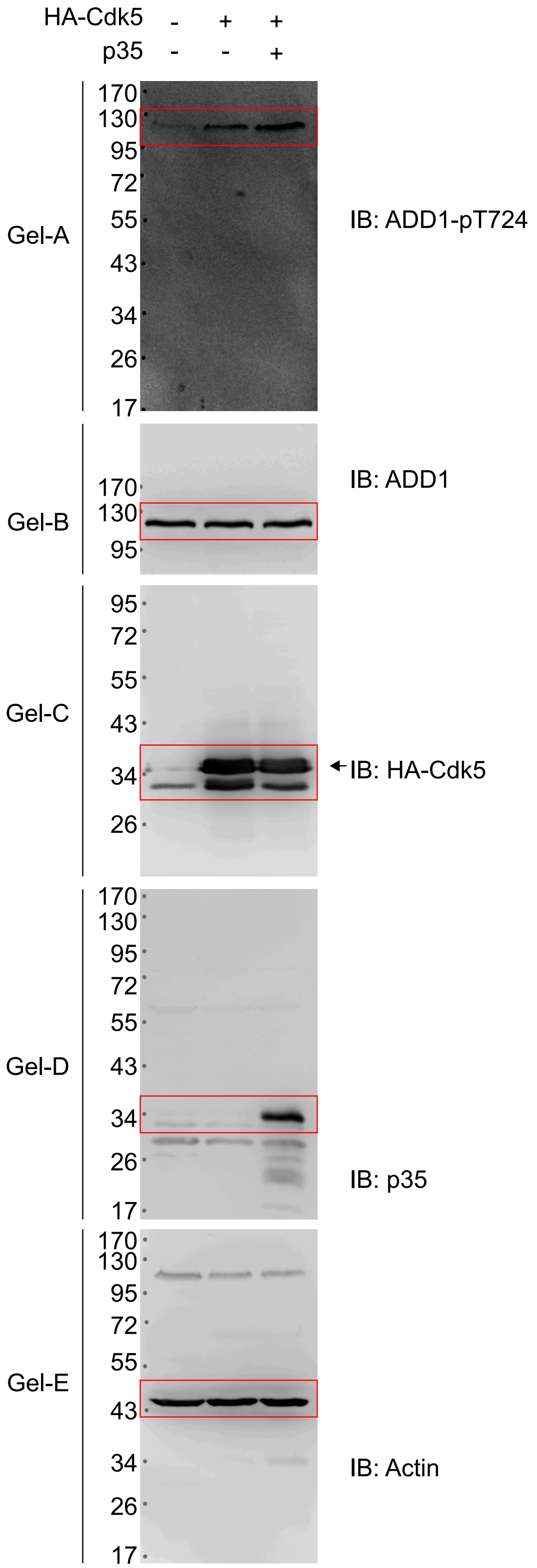


## Supple. Fig. 1(d)

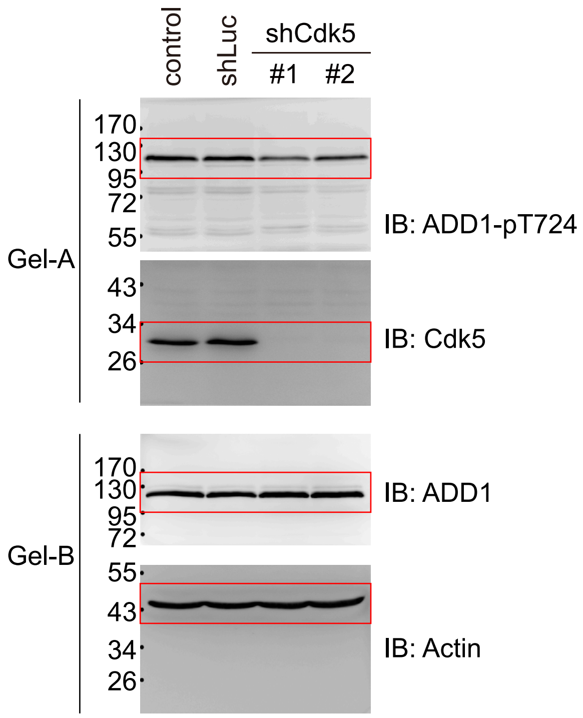

**Supple. Fig. 2(a)**

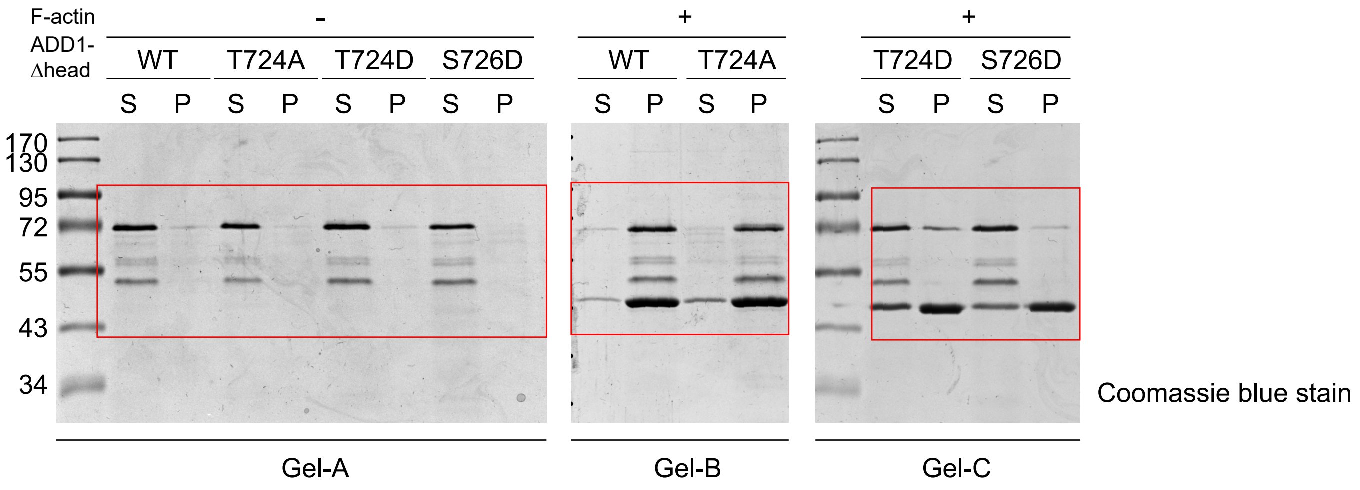

## Supple. Fig. 4(a)

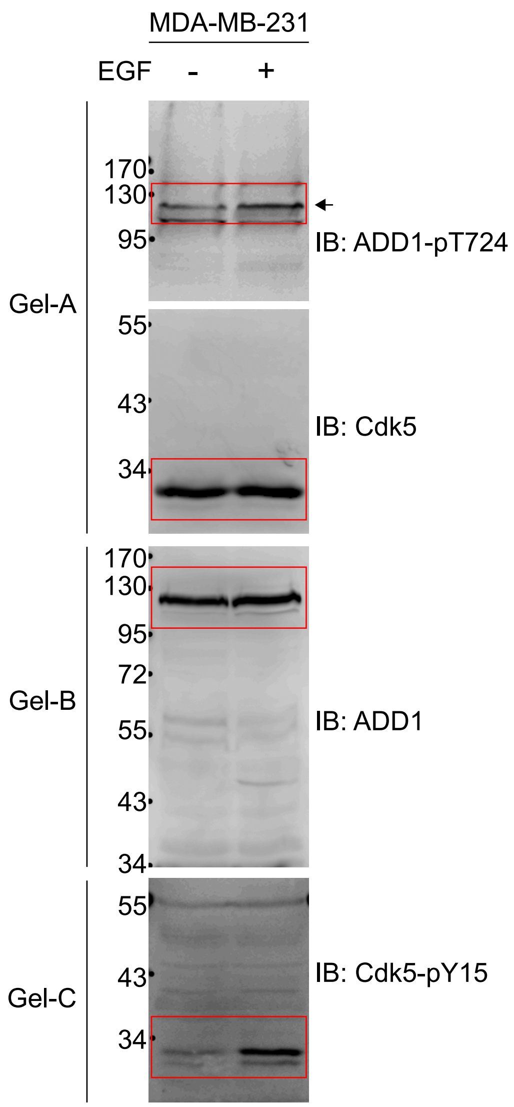


## Supple. Fig. 4(b)

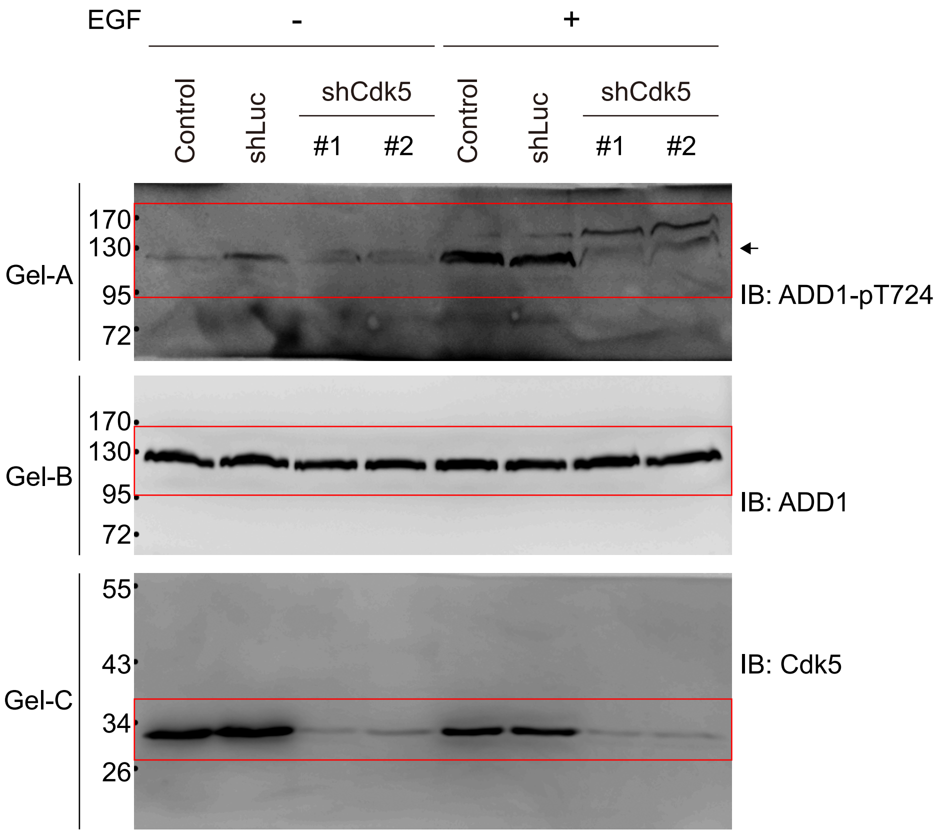

**Supple. Fig. 5(a)**

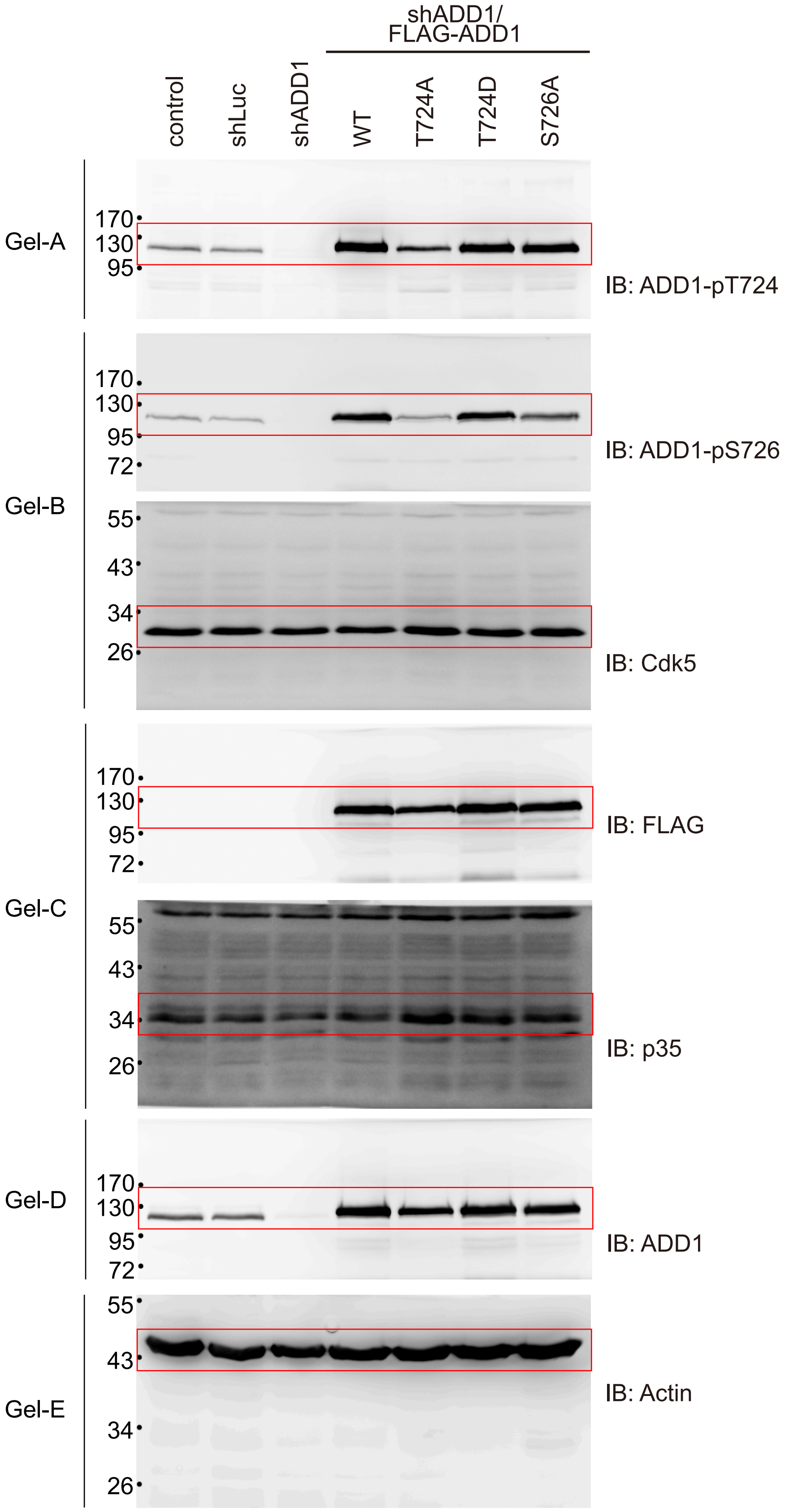

## Supple. Fig. 7(a)

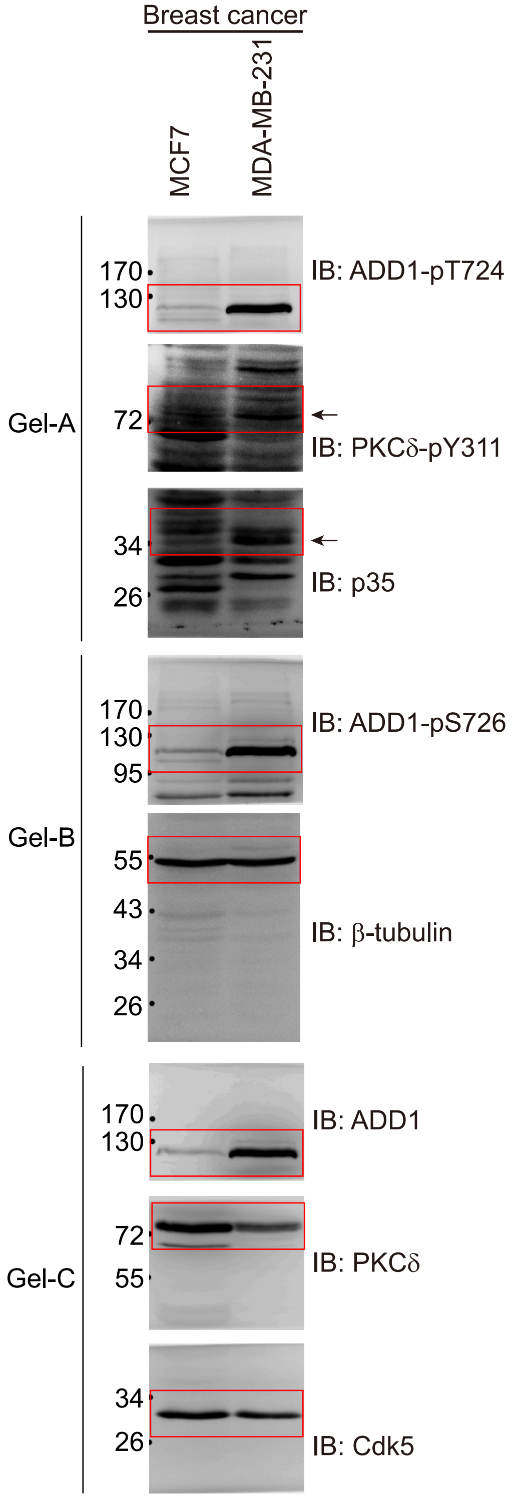


## Supple. Fig. 7(b)

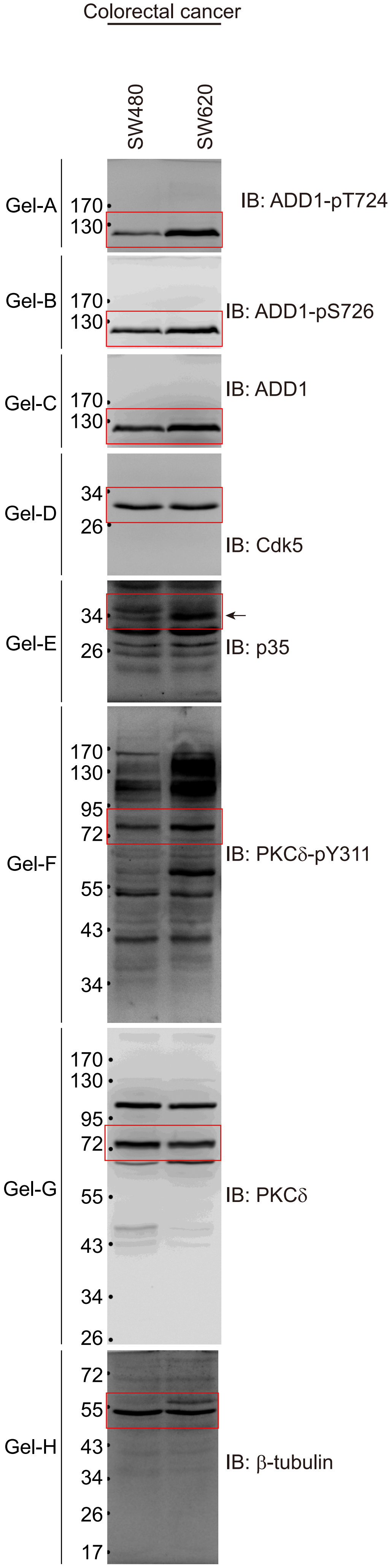

Supplement: Supplementary file 1 — Supplementary Information [file 41598_2019_50275_MOESM1_ESM.pdf]
